# Comparative evaluation of isoflurane and sevoflurane in avian patients

**DOI:** 10.14202/vetworld.2021.1067-1073

**Published:** 2021-05-04

**Authors:** R. R. Anjana, P. V. Parikh, J. K. Mahla, D. N. Kelawala, K. P. Patel, S. N. Ashwath

**Affiliations:** Department of Veterinary Surgery and Radiology, Anand Agricultural University, Anand, Gujarat, India

**Keywords:** anesthesia, avian patients, comparison, isoflurane, sevoflurane

## Abstract

**Background and Aim:**

Literature comparing the use of isoflurane and sevoflurane inhalation anesthetic agents in birds is scarce. This study aimed to evaluate the comparison of isoflurane and sevoflurane during induction, maintenance, and recovery of anesthesia in avian patients.

**Materials and Methods:**

In this study, 24 injured avian patients (n=24) were selected randomly and divided into four groups during kite flying festival. In the present study, isoflurane and sevoflurane were used as induction and maintenance anesthetic agents, with and without butorphanol tartrate premedication agent in all the birds. Different physiological parameters were evaluated, namely, cloacal temperature (°F), heart rate (beats/min), respiratory rate (breaths/min), and SpO_2_ (%) were recorded at 0, 10, 20 min, and at recovery time. The quality of anesthesia was assessed on the basis of induction time, quality of induction, production of analgesia, muscle relaxation, body reflexes, recovery time, quality of recovery, sitting, standing, and complete recovery time (CRT).

**Results:**

The mean±standard error value of induction time was 230.00±32.55, 280.00±25.29, 180.00±21.90, and 260.00±36.87 s, respectively, in Groups I, II, III, and IV. The feather plucking, pharyngeal, and toe pinching reflexes were noticed, when the birds were passing through the light plane of anesthesia during induction. Comparison of cloacal temperature at the time of recovery between Group-I versus Group-III revealed a significant difference (p<0.05). Comparison of mean respiratory rates at the time of recovery between Group-II versus Group-IV revealed a significant difference (p<0.05). Excellent quality of recovery was observed in all the groups of anesthetic protocols. Sitting, standing, and CRT were observed shortest in avian patients maintained with sevoflurane as compared to isoflurane.

**Conclusion:**

The quality of induction of anesthesia was rapid in avian patients when induced with sevoflurane as compared to isoflurane. Rapid onset of induction and recovery of anesthesia were found with sevoflurane followed by isoflurane. Induction and maintenance of anesthesia in avian patients with sevoflurane resulted in the lowest time required for sitting, standing, and CRT.

## Introduction

Avian is probably the most easily recognized of all animal species. There are many obvious differences in size, ranging from the hummingbird to the ostrich in the varying forms of the bill and in the color and profusion of the plumage occurring in the different species of birds [1]. Anesthesia is an important and challenging aspect of avian medicine and surgery. Birds have unique anatomical and physiological features that have an important impact on anesthesia [2]. The gaseous anesthetic agents used in modern practice include the fluorinated ethers isoflurane, sevoflurane, and desflurane. These modern agents have greatly improved the safety, rehabilitation, and applicability of general anesthesia [3].

General anesthesia in avian patients may induce by administration of either injectable or inhalation anesthetic agents. Injectable anesthetics may offer advantages such as low cost and minimal equipment. The use of ketamine as sole anesthetic in birds is not recommended because of poor muscle relaxation, myotonic contractions, opisthotonus, muscular tremors, and prolonged recoveries. Owls are more sensitive to ketamine-diazepam anesthetic combination. Abundant body fat was found to be an important factor in calculation of the proper dose of this combination [4].

Isoflurane continues to be a popular anesthetic agent for birds due to its relative safety, effectiveness, changes in the depth of anesthesia, and recovery can be easily and quickly controlled. Its faster induction and recovery are reported to have a sparing effect on cardiovascular function, cerebral blood flow, and its autoregulation and negligible metabolism make isoflurane useful in the anesthetic management of debilitated, aged, or exotic patients. Most of eliminated through the lungs with only a minute fraction metabolized in the liver [5]. Isoflurane provides good analgesia and adequate muscle relaxation. It was also a stable agent and resistant to metabolic breakdown. Therefore, it has a higher level of safety in those patients with compromised liver or kidney function [6]. Sevoflurane is a safe and versatile inhalation anesthetic agent as compared with currently available agents. Sevoflurane characteristics include inherent stability, low flammability, non-pungent odor, lack of irritation to airway passages, low blood gas solubility allowing rapid induction and emergence from anesthesia, minimal cardiorespiratory side effects, minimal end-organ effects, minimal effect on cerebral blood flow, low reactivity with other medications, and a vapor pressure and boiling point that enable delivery using standard vaporization techniques. As a result, sevoflurane has become one of the most widely used agents in its class [7].

Sevoflurane is an excellent, albeit more expensive option for bird anesthesia. The advantages of used sevoflurane versus isoflurane in birds include faster induction and recovery due to decreased blood and tissue solubility and smoother recoveries with less ataxia [8]. This study aimed to evaluate the comparison of isoflurane and sevoflurane during induction, maintenance, and recovery of anesthesia in avian patients.

## Materials and Methods

### Ethical approval

This study is a clinical work. So, it does not require ethical approval under the guidelines of CPCSEA.

### Study location and period

The study was conducted in the Department of Veterinary Surgery and Radiology, College of Veterinary Science and Animal Husbandry, AAU, Anand-388001 and Wildlife Care Centre (WCC), Ahmedabad-380054 from 16 August 2019 to 27 February 2020.

### Birds

Injured avian patients were captured and transported to the hospital by the volunteers, who were trained for handling of injured birds. Avian patients enrolled in the present study were having a huge variation in their body weight, ranging from 0.335 kg Domestic Rock Dove (*Columba livia domestica*) to 3.890 kg White Chinese Goose (*Anser cygnoides*).

### Study design

**Figure-1:**
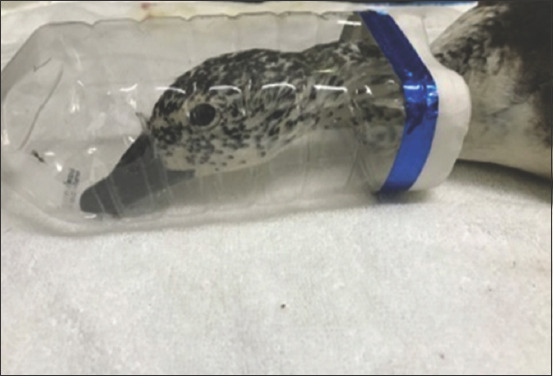
Avian patients induced by custom-made face mask.

**Figure-2:**
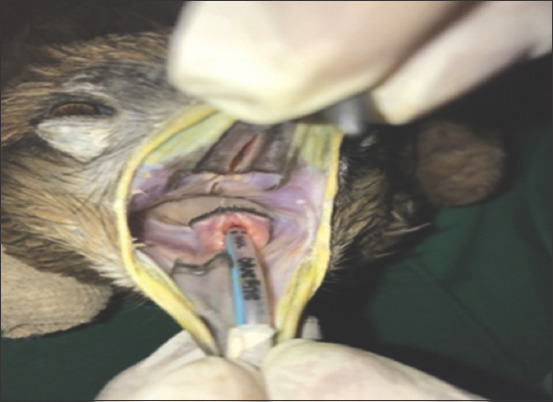
Kite intubated by uncuffed endotracheal tube.

In the present study, 24 injured avian patients (n=24) were selected and divided into four groups. In Group-I (n=6) butorphanol tartrate at 1.5 mg/kg, intramuscular (IM) was given 10 min before anesthesia followed by induction (3-4%) and maintenance (1-2.5%) with isoflurane. In Group-II (n=6), anesthesia was induced (3-4%) and maintained (1-2.5%) with isoflurane. In Group-III (n=6) butorphanol tartrate at 1.5 mg/kg, IM was given 10 min before anesthesia followed by induction (5-7%) and maintenance (3-4%) with sevoflurane. In Group-IV (n=6), anesthesia was induced (5-7%) and maintained (3-4%) with sevoflurane. Butorphanol tartrate was not administered in Groups II and IV as pre-anesthetic agents. Mask induction was performed in all birds using custom-made face masks (Figure-1). Endotracheal intubation with uncuffed endotracheal tube was performed immediately after induction (Figure-2). Physiological parameters, namely, cloacal temperature (°F), heart rate (beats/min), respiratory rate (breaths/min), and SpO_2_ (%) were recorded. The quality of anesthesia was assessed on the basis of induction time, quality of induction, production of analgesia, muscle relaxation, body reflex, recovery time (from the end of anesthesia to the first movement of avian patients.), quality of recovery, sitting time (from discontinuation of anesthesia to avian patients sit unassisted), standing time (from the end of anesthesia to avian patients stand unassisted), and complete recovery time (CRT) (from the end of anesthesia until avian patients resumes feeding and drinking).

The quality of induction was scored subjectively as follows: EXCELLENT: Assumed no struggling/avoidance behavior. VERY GOOD: Assumed minor avoidance behavior (random head and body movements), no vocalization. GOOD: Assumed purposeful attempts to move away from mask. FAIR: Assumed repeated escape attempts and vocalization. POOR: Assumed bird difficult to restrain, wing flapping, struggling, and vocalization.

The quality of recovery was scored subjectively as follows: EXCELLENT: Assumed sternal position with little or no struggle; walked without assistance or struggle; once standing; did not fall to sternal recumbency; minimal ataxia when walking. SATISFACTORY: Assumed sternal position with little or no struggle; premature standing without weakness in hind limbs; once standing, fall to sternal recumbency unlikely; slight ataxia. POOR: Some struggling; repeated attempts to move from lateral to sternal recumbency; premature standing with splayed and weak hind limbs; once standing, repeatedly falls to sternal recumbency; manual restraint required to avoid injury.

### Statistical analysis

The data generated were analyzed using a completely randomized design under SAS 9.3 software (Statistical Analysis System Institutes, Cary, NC, USA.). The data are presented as Mean±SE.

## Results and Discussion

Birds commonly get traumatic injuries by glass-coated thread (*Manja*) during the celebration of the kite flying festival in Gujarat. Before surgery, a detailed physical examination of injured avian patients was carried out. Out of 24 injured avian patients, 17 propatagial injury, 2 leg injury, 4 wing injury, and 1 crop fistula were observed and randomly selected for this study. Avian patients were stabilized by providing fluid therapy and supportive medications before anesthesia and surgical interventions.

Eye ointment or artificial tears should be used before pre-anesthetic agent administration in birds, especially those with kerato-conjuctivitis sicca, which prevent eye injuries [9]. A milder degree of sedation was observed after administration of butorphanol tartrate in birds. Butorphanol tartrate was not used as a premedication in Groups-II and IV, so struggling behavior was noticed during the initial phase of mask induction in Groups-II and IV. A very minute or no struggling effects were observed in Groups-I and III. All avian patients were induced safe and successful without any complications. Formation of salivation and mucus plug was not observed when avian patients were induced by isoflurane or sevoflurane and with or without premedication in avian patients.

**Figure-3:**
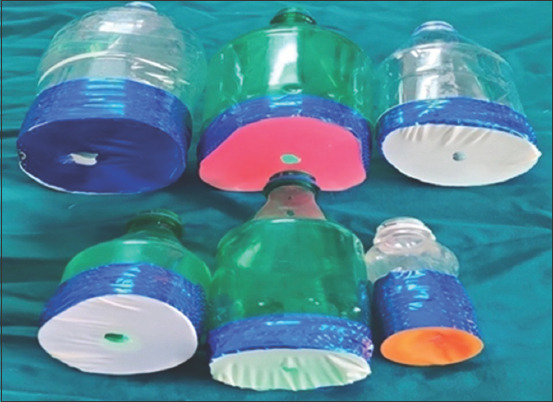
Custom-made face mask for birds.

The avian patients (n=24) covered under Groups-I, II, III, and IV were induced by inhalation anesthesia using custom-made face masks, which were prepared from clear plastic bottles and balloons (Figure-3). Custom-made masks provided an advantage of the desired fitting around the neck of birds, effective induction, minimize anesthetic contamination, adequate monitoring opportunities, and cheaper in cost. The Mask induction technique was found to be successful and safe in all birds. The present findings of mask induction were observed by Mer *et al*. [10].

The mean±standard error (SE) time of induction of anesthesia was found to be the lowest in the avian patients under Group-III (180.00±21.90 s) followed by, in ascending order, those of -I (230.00±32.55 s), -IV (260.00±36.87 s), and -II (280.00±25.29 s). The details of the results are presented in Table-1. The Mean±SE time of induction in avian patients was found to be comparatively lower under Group-III (180.00±21.90 s) versus Group-I (230.00±32.55 s), with the difference being non-significant. Similarly, the Mean±SE time of induction in avian patients was found to be comparatively lower under Group-IV (260.00±36.87 s) versus -II (280.00±25.29 s), with non-significant difference. These observations clearly indicated that better and shorter time of induction of anesthesia could be achieved using sevoflurane as compared to isoflurane. Similar findings are reported in crested serpent eagles by Chan *et al*. [11]. The advantages of using sevoflurane versus isoflurane in avian patients produced faster induction due to decrease blood and tissue solubility [8].

**Table-1:** Time of induction of anesthesia in avian patients (n=24).

Groups (n=6, each)	Induction time* (s) (Mean±SE)
Group-I	230.00±32.55^bac^
Group-II	280.00±25.29^a^
Group-III	180.00±21.90^dc^
Group-IV	260.00±36.87^ba^

Least significant difference: 71.741.

* Means bearing different superscripts (a, b, c, and d) in a column differ significantly (p<0.05)

**Figure-4:**
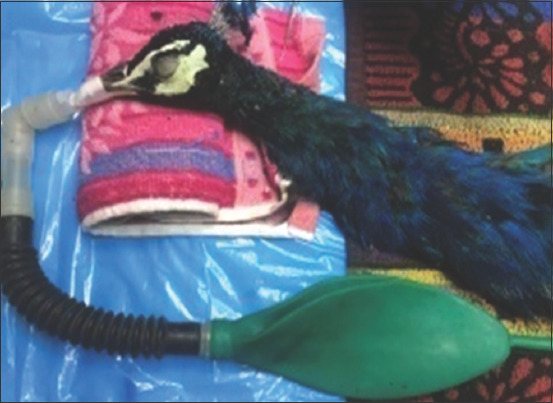
Maintenance of anesthesia by inhalation anesthetic agents in Indian peacock.

The feather plucking, pharyngeal, and toe pinching reflexes were noticed when the birds were passing through the light plane of anesthesia using inhalation agents for induction. The quality of analgesia achieved better in the butorphanol tartrate pre-medicated avian patients as compared to without butorphanol tartrate premedication. No befitting literature could be traced while screening the reference to substantiate the present findings. The details of the results are presented in Table-2. Avian patients were safe, successfully and without any complication maintained (Figure-4) by isoflurane and sevoflurane.

**Table-2:** Grading of quality of induction of anesthesia in avian patients (n=24).

Quality	Percent of induction	Inhalation induction				

		Isoflurane		Pooled value	Sevoflurane		Pooled value
	
		Group-I	Group-2		Group-3	Group-4	
		I	II		III	IV	
Excellent	20.83 (05)	33.33 (02)	-	16.66 (02)	50.00 (03)	-	25.00 (03)
Very good	4.16 (01)	16.66 (01)	-	8.33 (01)	-	-	-
Good	20.83 (05)	33.33 (02)	16.66 (01)	25.00 (03)	33.33 (02)	-	16.66 (02)
Fair	16.66 (04)	-	33.33 (02)	16.66 (02)	-	33.33 (02)	16.66 (02)
Poor	37.50 (09)	16.66 (01)	50.00 (03)	33.33 (04)	16.66 (01)	66.66 (04)	41.66 (05)
Total (n=6, each)	100.00 (24)	6	6	-	6	6	-

Figures in parenthesis indicate number of birds

Comparison of cloacal temperature at the time of recovery between Group-I and Group-III revealed a significant difference (p<0.05), where cloacal temperature remains higher in isoflurane protocols as compared to the sevoflurane protocols. The cloacal temperature decreased gradually throughout anesthesia in all the groups, despite supplemental heat provided by heating pad. The findings are supported by the similar results of Botman *et al*. [12]. Cloacal temperature gradually declined throughout anesthesia in avian patients maintained with isoflurane and sevoflurane anesthetic agents. The present findings were corroborated well with the observations of Deori *et al*. and Isler *et al*. [13,14]. The details of the results are presented in Table-3. The Mean±SE value of heart rates in avian patients found to be differing non-significantly at the time of recovery in Group-I ­versus -III and Group-II versus -IV. In the present study, the heart rate in the avian patients declined gradually throughout the maintenance of anesthesia using isoflurane and sevoflurane. These findings are supported and similar results reported by Deori *et al*. [13]. The details of the results are presented in Table-4.

**Table-3:** Cloacal temperature (°F) following anesthesia using different protocols in avian patients (n=24).

Groups (code) (n=6, each)	Time intervals (Mean±SE)			

	0 min	10 min	20 min	Recovery
Group-I	103.91±0.29^bac^	103.48±0.35^ba^	103.01±0.29^a^	101.16±0.28^ba^
Group-II	104.46±0.58^a^	103.81±0.61^a^	103.25±0.64^a^	101.38±0.31^a^
Group-III	103.01±0.21^bc^	102.46±0.23^bc^	101.63±0.20^bc^	100.05±0.24^edc^
Group-IV	104.30±0.56^a^	103.40±0.63^bac^	102.36±0.54^bac^	100.75±0.36^bac^

Least significant difference: 0 min=1.24; 10 min=1.32; 20 min=1.22; R=0.95. Means bearing different superscripts (a, b, c, and d) in a column differ significantly (p<0.05)

**Table-4:** Heart rates (beats/min) during anesthesia using different protocols in avian patients (n=24).

Groups (n=6, each)	Time interval (Mean±SE)			

	0 min*	10 min*	20 min*	Recovery*
Group-I	108.00±7.59^c^	104.66±7.43^a^	108.00±7.99^a^	118.00±7.95^a^
Group-II	156.16±6.46^ba^	126.33±5.76^a^	121.50±5.45^a^	136.00±5.62^a^
Group-III	108.16±16.47^c^	103.33±16.21^a^	99.50±16.22^a^	112.66±16.85^a^
Group-IV	136.00±5.51^bac^	102.16±5.64^a^	96.33±5.77^a^	105.00±5.95^a^

Least significant difference: 0 min=38.05, 10 min=37.51, 20 min=36.16 and R=37.89.

* Means bearing different superscripts (a, b, c, and d) in a column differ significantly (p<0.05)

Comparison of Mean±SE value of respiratory rates at the time of recovery between Group-II and Group-IV revealed a significant difference (p<0.05), where the respiratory rate remained higher in isoflurane groups as compared to sevoflurane groups. Declined respiration rate in avian patients using isoflurane in the present study could be due to the respiratory depressant activity as reported by Chan *et al*. [11] and Joyner *et al*. [15] in crested serpent eagle and bald eagle, respectively. The details of the results are presented in Table-5.

**Table-5:** Respiratory rate (breaths/min) during anesthesia using different protocols in avian patients.

Groups (n=6, each)	Time interval (Mean±SE)			

	0 min*	10 min*	20 min*	Recovery*
Group-I	25.83±2.31^b^	23.50±2.56^bac^	20.83±2.02^bac^	22.50±1.14^ba^
Group-II	32.50±1.58^a^	27.83±2.35^a^	25.00±2.32^a^	24.50±1.25^a^
Group-III	23.00±1.84^b^	20.50±2.10^bc^	16.66±1.56^dc^	20.33±0.98^bc^
Group-IV	27.50±1.54^ba^	23.33±1.28^bac^	19.00±1.36^bdc^	21.16±0.94^bc^

Least significant difference: 0 min=5.52, 10 min=5.50, 20 min=5.09 and R=3.29.

* Means bearing different superscripts (a, b, c, and d) in a column differ significantly (p<0.05)

Saturation pressure of oxyhemoglobin (SpO_2_) indicated as a fraction of oxygen saturated hemoglobin relative to total hemoglobin in the blood, which helped the anesthesiologist to constantly monitor the avian patient and to provide emergency management so as to save the life of the patient. However, the difference between Mean±SE percent SpO_2_ found in the present study in the avian patients under isoflurane and sevoflurane protocols at the time of recovery was non-significant. The Mean±SE percent SpO_2_ was recorded higher in the maintenance of anesthesia with sevoflurane as compared to isoflurane, probably due to the low blood gas solubility of sevoflurane. The present findings are supported well by the similar observations reported by Degernes [8] in avian patients. The details of the results are presented in Table-6.

**Table-6:** SpO_2_ (%) during anesthesia using different protocols in avian patients (n=24).

Groups (n=6, each)	Time interval (Mean±SE)			

	0 min*	10 min*	20 min*	Recovery*
Group-I	85.66±0.49^ed^	81.16±1.19^d^	75.66±0.71^d^	85.50±0.95^bac^
Group-II	89.66±0.88^b^	85.00±0.44^ba^	81.66±0.49^ba^	87.16±0.79^a^
Group-III	87.33±0.66^cd^	83.33±1.96^bcd^	80.16±1.49^b^	86.50±1.20^ba^
Group-IV	91.83±0.79^a^	88.00±0.51^a^	84.16±1.77^a^	88.50±1.43^a^

Least significant difference: 0 min=2.01, 10 min=3.04, 20 min=3.48 and R=3.27.

* Means bearing different superscripts (a, b, c, and d) in a column differ significantly (p<0.05)

**Figure-5:**
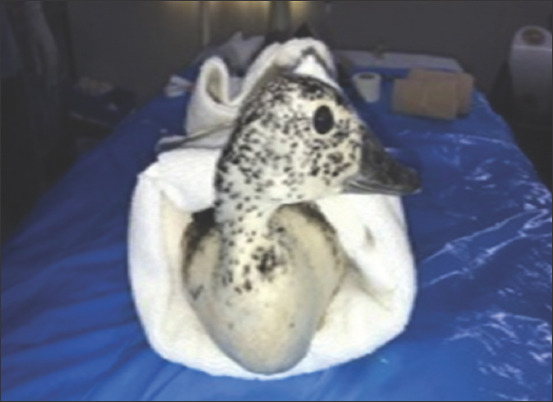
Combed duck successfully recovered from inhalation anesthesia.

The Mean±SE value of recovery time was found to be the shorter in Group-IV (140.00±12.64 s), followed by, in an ascending manner, Groups-III (160.00±29.66 s), -II (190.00±28.63 s), and -I (220.00±42.89). The details of the results are presented in Table-7. Comparison of the time of recovery between Group-I versus -III and Group-II versus -IV was revealed non-significant differences. Birds under sevoflurane protocols had a rapid onset of recovery as compared to isoflurane protocols. The present findings are comparable with the similar observations reported by Joyner *et al*. [15] in the domestic pigeon and Chan *et al*. [11] in the crested serpent eagle. Maintaining the anesthesia using sevoflurane as compared to isoflurane was found to be advantageous with faster and smooth recovery due to lower blood and tissue solubility as opined by Degernes [8]. Adequate analgesia and muscle relaxation was reported in all the avian patients during surgical interventions. The present findings were corroborated well with the observations of Miller and Buttrick [6]. Excellent quality of recovery (Figure-5) was recorded in all the birds. The details of the results are presented in Table-8.

**Table-7:** Time of recovery of anesthesia in avian patients (n=24).

Groups (n=6, each)	Recovery time*(s) (Mean±SE)
Group-I	220.00±42.89^bdac^
Group-II	190.00±28.63^bdc^
Group-III	160.00±29.66^dc^
Group-IV	140.00±12.64^d^

Least significant difference: 90.27.

* Means bearing different superscripts (a, b, c, and d) in a column differ significantly (p<0.05)

**Table-8:** Quality of recovery of avian patients from anesthesia (n=24).

Group no. (n=6 each)	I	II	III	IV	Percent recovery
Quality					
Excellent	100.00 (06)	100.00 (06)	100.00 (06)	100.00 (06)	100.00 (24)
Satisfactory	-	-	-	-	-
Poor	-	-	-	-	-

Figures in parenthesis include number of birds

The shortest sitting time was observed in birds which anesthetized with Group-IV (290.00±28.63 s). The details of the results are presented in Table-9. The Mean±SE value of sitting time was found to be differing non-significantly between Group-II versus -IV and Group-I versus -III. Shortest standing time observed in birds under Group-IV (470.00±28.63 s) followed by Groups-II (510.00±55.31 s), -III (560.00±42.89 s), and -I (590.00±39.24 s). The details of the results are presented in Table-10. The Mean±SE values of standing time of anesthesia differing were non-significantly between anesthetic agents of isoflurane and sevoflurane groups. Sitting and standing time was observed to be the shortest in sevoflurane groups as compared to isoflurane groups.

**Table-9:** Sitting time of anesthesia in avian patients (n=24).

Groups (n=6, each)	Sitting time* (s) (Mean±SE)
Group-I	380.00±42.89^dc^
Group-II	340.00±36.87^d^
Group-III	380.00±25.29^dc^
Group-IV	290.00±28.63^d^

Least significant difference: 118.54.

* Means bearing different superscripts (a, b, c, and d) in a column differ significantly (p<0.05)

**Table-10:** Standing time in avian patients under different anesthesia (n=48).

Groups (n=6, each)	Standing time* (s) (Mean±SE)
Group-I	590.00±39.24^bcd^
Group-II	510.00±55.31^cd^
Group-III	560.00±42.89^bcd^
Group-IV	470.00±28.63^d^

Least significant difference: 163.32.

* Means bearing different superscripts (a, b, c, and d) in a column differ significantly (p<0.05)

The time required to recover the birds fully from the anesthesia is considered as a CRT. The shortest Mean±SE value of CRT was observed in avian patients anesthetized under Group-IV (18.16±1.35 min) and the longest CRT was observed in birds under Group-I (25.83±1.24 min). The details of the results are presented in Table-11. The Mean±SE value of CRT of anesthesia differing was non-significantly between anesthetic agents of isoflurane and sevoflurane groups.

**Table-11:** Complete recovery time of anesthesia in avian patients (n=48).

Groups (n=6, each)	Complete recovery time* (min) (Mean±SE)
Group-I	25.83±1.24^dc^
Group-II	20.66±1.38^e^
Group-III	22.50±1.89^de^
Group-IV	18.16±1.35^e^

Least significant difference: 4.60.

* Means bearing different superscripts (a, b, c, and d) in a column differ significantly (p<0.05)

## Conclusion

Butorphanol tartrate achieved smooth, rapid, and safe induction in avian patients. The custom-made face mask for inhalation anesthesia seemed to be very convenient, effective, and cheaper for avian patients with varying beak sizes. Avian patients induced and maintained with sevoflurane anesthesia (Group-4) reported lowest sitting, standing, recovery, and CRT among all groups of anesthesia, where 100% of birds showed safe and excellent quality of recovery, hence proved to be the best anesthesia among all anesthetic protocols.

## Authors’ Contributions

PVP, JKM, and RRA participated equally in the study plan, design, and drafted the manuscript. DNK, KPP, and SNA contributed to surgical operations. PVP, JKM, and RRA drafted and corrected the manuscript. All authors read and approved the final manuscript.
